# Fbxo41 Promotes Disassembly of Neuronal Primary Cilia

**DOI:** 10.1038/s41598-019-44589-2

**Published:** 2019-06-03

**Authors:** Cillian R. King, Ana R. A. A. Quadros, Anaël Chazeau, Ingrid Saarloos, Anne Jolien van der Graaf, Matthijs Verhage, Ruud F. Toonen

**Affiliations:** 1 Department of Functional Genomics, Center for Neurogenomics and Cognitive Research, VU University Amsterdam and VU Medical Center, 1081 HV Amsterdam, The Netherlands; 2 Department of Clinical Genetics, Center for Neurogenomics and Cognitive Research, VU University Amsterdam and VU Medical Center, 1081 HV Amsterdam, The Netherlands; 3 Cell Biology, Faculty of Science, Utrecht University, 3584 CH Utrecht, The Netherlands

**Keywords:** Neuronal physiology, Molecular neuroscience, Development of the nervous system

## Abstract

Neuronal primary cilia are signaling organelles with crucial roles in brain development and disease. Cilia structure is decisive for their signaling capacities but the mechanisms regulating it are poorly understood. We identify Fbxo41 as a novel Skp1/Cullin1/F-box (SCF) E3-ligase complex subunit that targets to neuronal centrioles where its accumulation promotes disassembly of primary cilia, and affects sonic hedgehog signaling, a canonical ciliary pathway. Fbxo41 targeting to centrioles requires its Coiled-coil and F-box domains. Levels of Fbxo41 at the centrioles inversely correlate with neuronal cilia length, and mutations that disrupt Fbxo41 targeting or assembly into SCF-complexes also disturb its function in cilia disassembly and signaling. Fbxo41 dependent cilia disassembly in mitotic and post-mitotic cells requires rearrangements of the actin-cytoskeleton, but requires Aurora A kinase activation only in mitotic cells, highlighting important mechanistical differences controlling cilia size between mitotic and post-mitotic cells. Phorbol esters induce recruitment of overexpressed Fbxo41 to centrioles and cilia disassembly in neurons, but disassembly can also occur in absence of Fbxo41. We propose that Fbxo41 targeting to centrosomes regulates neuronal cilia structure and signaling capacity in addition to Fbxo41-independent pathways controlling cilia size.

## Introduction

Primary cilia are antennae-like sensory organelles that project from the plasma membrane of a wide variety of cells, including neurons. They are composed of a microtubule-based core structure that elongates from a membrane-anchored modified centriole, the basal body^1^. Both primary cilia membrane and cytosol content are isolated from the rest of the cell. This allows cilia to concentrate several signaling pathways, and act as a sensory organelle for extracellular and intracellular cues^2^. For example, the Sonic hedgehog (Shh) cascade, a key signaling pathway during brain development, has been shown to depend on ciliary integrity^3,4^. Mutations disrupting ciliary structure can lead to debilitating disorders, referred to as ciliopathies, which have pleiotropic clinical features^5–7^. Among the 87 genes associated with ciliopathies, 77 genes have been linked to neurological deficits in humans^8^, such as altered brain anatomy, obesity, and intellectual disability^9^.

Cilia structure is dynamically modulated in both mitotic cells and neurons, with important functional consequences. As such, cilia assembly and disassembly are highly regulated processes, and can be induced by several signaling cues^10^ in mitotic cells. The most well-studied cue is cell cycle entry where cilia disassemble before cell division and re-assemble after the end of the cell cycle^11–13^. One canonical mechanism of ciliary disassembly in mitotic cells, is through the centrosomal kinase Aurora A: activation of Aurora A leads to activation of HDAC6, which deacetylates and destabilizes axonemal microtubules^14^. Primary cilia are also present in post-mitotic cells, such as neurons. Neuronal cilia structure is dynamic, and depends on developmental stage and final layer position of the cell^15,16^. In migrating cortical interneurons, cilia length is highly dynamic^17^ and cilia of olfactory neurons in *C*. *elegans* remodel in a sensory signaling-dependent manner^18^. This implies the presence of machinery that senses extracellular cues and modulates ciliary architecture. So far, however, the mechanisms through which neuronal primary cilia length is regulated remain elusive.

One powerful modulatory system to control cilia structure and function is the ubiquitin proteasome system (UPS). The UPS selectively modulates the cellular protein pool to temporally and spatially control cellular activities. UPS components have been shown to accumulate at the centrosome^19–21^, and are able to control ciliary length^22,23^. F-box proteins are substrate binding adaptors of a Skp1/Cullin1/F-box (SCF) E3-ligase complex^24^ that confer selectivity to the UPS by selecting the target of ubiquitination. Fbxo41 is a brain enriched F-box protein, with high expression in hippocampal neurons, where it accumulates in centrioles from which primary cilia emanate^25^, making it a prime target for regulating neuronal primary cilia.

In this study we show that Fbxo41 assembles into an SCF complex, targets to neuronal centrioles, and its accumulation promotes disassembly of primary cilia. Fbxo41 requires its Coiled-coil and F-box domains for targeting to centrioles. Centriolar Fbxo41 levels show strong inverse correlation with cilia length, but not in mutants with disrupted Fbxo41-Skp1 interaction. We show, for the first time, that neurons treated with the phorbol ester PDBU, but not canonical network-activity modulators (Gabazine, APV, DNQX or TTX) have shorter cilia and increased centrosomal Fbxo41 expression. However, ciliary disassembly induced by PDBU also occurs in absence of Fbxo41. The effect of Fbxo41 in cilia disassembly in mitotic cells can be rescued by inhibiting the canonical Aurora A pathway, or perturbating actin dynamics by cytochalasin D. The latter compound also prevents Fbxo41-dependent cilia shortening in neurons. Finally, we show that Fbxo41 disturbs Shh signaling, a prominent ciliary pathway. We propose a mechanism where neurons can shorten their cilia by regulating centriolar levels of Fbxo41, which affects ciliary signaling capacity.

## Results

### Fbxo41 is an SCF-complex subunit that targets to neuronal centrioles

**Figure 1:**
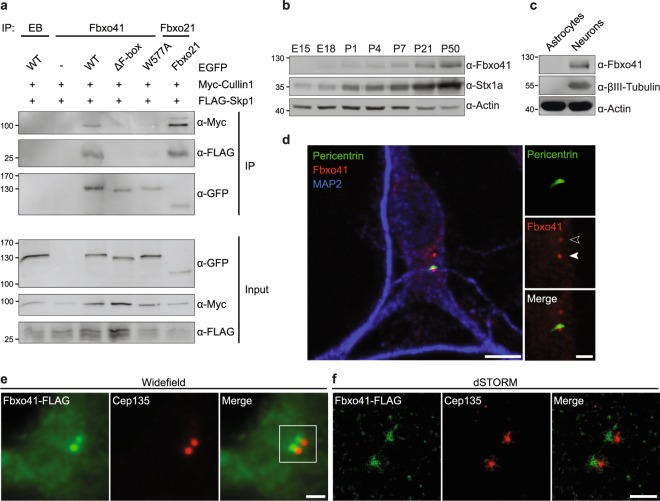
Fbxo41 assembles into SCF-complexes and targets to centrioles. (**a**) Fbxo41 assembles into SCF-complexes. HEK293T cells were transfected with the indicated constructs and subjected to immunoprecipitation with empty beads (EB), Fbxo41 or Fbxo21 antibody. Deleting (ΔF-box) or mutating (W577A) Fbxo41’s F-box domain prevented SCF-complex assembly. Fbxo21 was included as a positive control. Gel was cropped for clarity (full length blot available in Supplementary Fig. S5). (**b**) Fbxo41 is increasingly expressed in brain throughout development. Mouse brains were extracted at the indicated ages and immunoblotted with the indicated antibodies. Syntaxin1a was included as a positive control^62^. Gel was cropped for clarity (full length blot available in Supplementary Fig. S5). (**c**) Fbxo41 is expressed in neurons and not in astrocytes. High-density astrocyte or neuronal cultures were lysed at DIV11 and subjected to immunoblotting. βIII-tubulin was included to demonstrate the presence of primary cortical neurons. Gel was cropped for clarity (full length blot available in Supplementary Fig. S5). (**d**) Example of a primary hippocampal neuron fixed at DIV15 and immunostained with Fbxo41 (red), Pericentrin (green) and MAP2 (blue) antibodies. Endogenous Fbxo41 was observed in enrichments in the soma (open arrowhead). In a subset of neurons (such as this example), Fbxo41 was enriched at the pericentriolar region as indicated by Pericentrin (arrowhead). Scale bars, 5 and 2 μm. (**e**) Widefield fluorescence image of a primary DIV15 hippocampal rat neuron expressing Fbxo41^WT^-FLAG and immunostained with antibodies for FLAG (green) and Cep135 (red). Imaging was performed with FLAG-tagged Fbxo41^WT^ due to antibody incompatibility. Scale bar, 1 μm. (**f**) Super-resolution dSTORM image of boxed region in (**e**). Scale bar, 500 nm.

Generally, F-box proteins are modular substrate binding adaptors of a Skp1/Cullin1/F-box (SCF) E3-ligase complex^24^. Since this has not been established for every F-box protein family member^26–28^, we tested whether Fbxo41 assembles into a SCF-complex by expressing EGFP- or FLAG-tagged Fbxo41, Skp1 and Cullin1 in HEK293T cells, and performing immunoprecipitation experiments. Indeed, Fbxo41 associated with Skp1 and Cullin1, albeit less efficiently than Fbxo21 which was included as a positive control. Deleting (Fbxo41^ΔF-box^) or mutating (Fbxo41^W577A^) the F-box domain abolished these interactions, confirming that, like other F-box proteins, Fbxo41 assembles into SCF-complexes via an essential F-box domain (Fig. 1a and Supplementary Fig. S1).

In the brain Fbxo41 is exclusively expressed in neurons and Fbxo41 levels increase with age in the cerebellum^25^. We confirmed that Fbxo41 expression increases in whole-brain lysates throughout development (Fig. 1b), and show that Fbxo41 is expressed in cultured neurons, but not in astrocytes (Fig. 1c). Skp1 and Cullin1 are enriched at centrosomes of dividing cells where their ubiquitin-ligase activity is essential for many centrosomal functions controlling cell division^20,29–31^. Although our antibodies did not allow immunoprecipitation of sufficient amounts of Fbxo41 from brain lysate (Supplementary Fig. S1), we could assess the presence of Fbxo41 at centrosomes in primary hippocampal neurons. Endogenous Fbxo41 located throughout the cytosol with distinctive enrichments in the soma, which co-localized with the pericentriolar protein Pericentrin (Fig. 1d), but Fbxo41-EGFP did not colocalize with several other somatic organelles (Supplementary Fig. S2). Super-resolution stochastic optical reconstruction microscopy (STORM) revealed that Fbxo41-EGFP resides adjacent to centrosomal protein 135 (Cep135), a centriole proximal-end protein^32–34^ (Fig. 1e,f). Hence, Fbxo41 is a neuronal protein that targets to centrioles.

### Centrosomal targeting of Fbxo41 requires its F-box and coiled-coil domains

**Figure 2:**
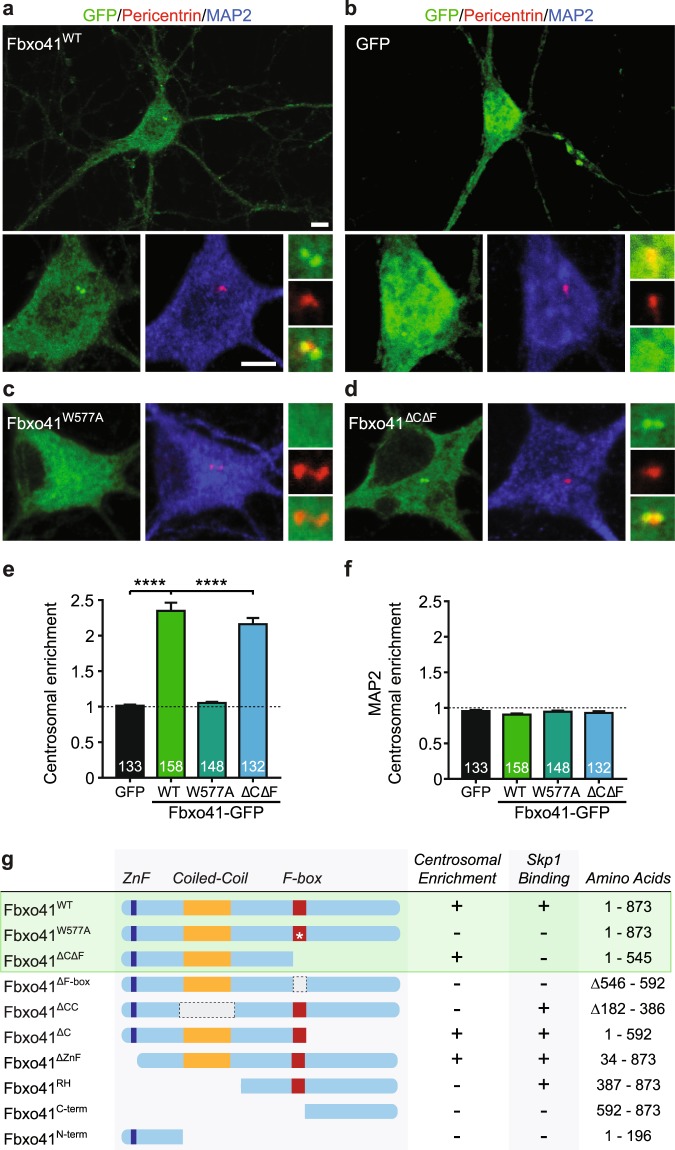
Fbxo41 is enriched in neuronal centrioles. (**a**–**d**) Primary hippocampal neurons were infected at DIV9 with GFP or the indicated Fbxo41 mutants, and fixed at DIV14. Centrioles were marked with Pericentrin antibody (red), neurons with MAP2 antibody (blue) and EGFP-Fbxo41 mutants with GFP (green). Fbxo41^WT^ (**a**), and Fbxo41^ΔCΔF^ (**d**) were enriched at pericentrin-positive centrioles, whereas GFP (**b**), and Fbxo41^W577A^ (**c**) were not. Scale bars, 5 μm. (**e**) Ratio of centrosomal over cytoplasmatic intensity was quantified for each Fbxo41 mutant and GFP. Fbxo41^WT^ and Fbxo41^ΔCΔF^ have a significantly higher enrichment at centrosomes, compared to GFP and Fbxo41^W577A^. (**f**) As a control, the ratio of centrosomal over cytoplasmatic intensity of MAP2 was quantified in the same neurons. MAP2 does not enrich in the centrosomes, indicating that the centrosomal enrichment of Fbxo41 is specific to this protein. (**g**) Scheme of EGFP-fused Fbxo41 mutants used for domain mapping. Colored boxes depict predicted domains: Zinc-finger (ZnF, dark blue), Coiled-coil (orange) and F-box domain (red). White asterisk represents W577A mutation. Centrosomal enrichment of the first three mutants (Fbxo41^WT^, Fbxo41^W577A^ and Fbxo41 ^ΔCΔF^, inside the green box) was tested in neurons and HEK293T cells, whereas the other mutants were studied in HEK293T cells (see Supplementary Fig. S3). Data from 4 experimental weeks are represented as mean ± SEM. ****p < 0,0001 Kruskal-Wallis Test, followed by a post-hoc Dunn’s multiple comparisons test.

The closest homologue of Fbxo41, ZNF365, is a brain-enriched zinc finger protein that targets to centrosomes via its Coiled-coil domain^35^. In addition, Fbxo41 was shown before to require its Coiled-coil domain to target to centrosomes^25^. We further characterized the Fbxo41 domains required for centrosomal targeting by quantifying centrosomal enrichment of several other Fbxo41 mutants in neurons and HEK293T cells (Fig. 2 and Supplementary Fig. S3). Indeed, in HEK293T cells mutants with a deleted Coiled-coil domain (Fbxo41^ΔCC^, Fbxo41^RH^, Fbxo41^C-term^ and Fbxo41^N-term^) did not target to centrosomes, regardless of an intact F-box domain, indicating that the Coiled-coil domain is indeed necessary for Fbxo41 centrosomal targeting (Fig. 2g and Supplementary Fig. S3). However, mutants with a deleted (Fbxo41^ΔF-box^) or mutated (Fbxo41^W577A^) F-box domain were also diffusely located throughout the cytoplasm (Supplementary Fig. S3). Together, this suggests that both the Coiled-coil and the F-box domains are required for centrosome targeting. A truncation containing only the right half of the protein (Fbxo41^RH^), which includes the F-box domain but lacks the Coiled-coil domain, failed to target to centrosomes (Supplementary Fig. S3) showing that the F-box domain is not sufficient for centrosomal targeting. Unexpectedly, a mutant that lacked the F-box domain and the C-terminus (Fbxo41^ΔCΔF^) did target to centrosomes. Finally, deletion of the zinc-finger domain (Fbxo41^ΔZnF^) did not affect centrosomal targeting.

To verify Fbxo41 centrosome-targeting domains in neurons, we assessed the co-localization of several Fbxo41 mutants with pericentrin, an endogenous centrosomal protein (Fig. 2). We expressed Fbxo41^WT^ (Fig. 2a), GFP (Fig. 2b), and Fbxo41 mutants (Fig. 2c,d) and quantified their centrosomal enrichment in primary hippocampal neurons (Fig. 2e). Both Fbxo41^WT^, and Fbxo41 lacking the F-box domain and the C-terminus of the protein (Fbxo41^ΔCΔF^) were enriched at the centrosome; in contrast, Fbxo41 with a mutated F-box domain (Fbxo41^W577A^) was diffusely located throughout the cytoplasm, corroborating our findings in HEK293T cells. The neuronal protein MAP2 did not accumulate at the centrosome in neurons expressing GFP or Fbxo41 constructs (Fig. 2f), indicating that Fbxo41 accumulation at the centrosome is specific.

Collectively, these results are compatible with a model in which both Fbxo41’s Coiled-coil and F-box domains are required for centrosomal targeting. The C-terminal prevents centrosomal targeting of Fbxo41, possibly through steric hindrance of the Coiled-coil domain, that can be released by the F-box domain.

### Fbxo41 promotes disassembly of primary cilia

**Figure 3:**
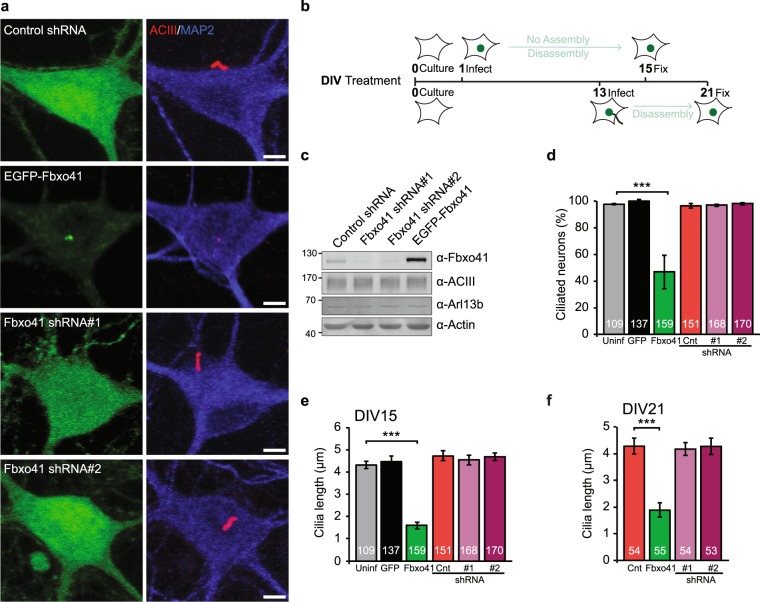
Fbxo41 promotes disassembly of primary cilia. (**a**) Typical examples of images used to quantify cilia length. (**b**) Primary hippocampal neurons were infected with the indicated constructs at DIV1 or 13, and fixed at DIV15 or 21, and immunostained with GFP (green), ACIII (red) and MAP2 (blue) antibodies. (**c**) Immunoblot of primary cortical neurons infected with the indicated constructs at DIV1 and lysed at DIV16. Manipulating Fbxo41 expression does not alter ACIII or Arl13b expression levels. Note the diffuse ACIII band is caused by variable glycosylation^63,64^. Gel was cropped for clarity (full length blot available in Supplementary Fig. S5). (**d**) Percentage of ciliated neurons infected at DIV 1 and fixed at DIV 15. Fbxo41 accumulation, but not depletion, significantly affects the percentage of ciliated neurons. Data from three independent experiments. (**e**) Quantification of cilia length from neurons in (**d**). (**f**) Cilia length in neurons infected with the indicated constructs at DIV 13 and fixed at DIV21. Fbxo41 promotes cilia disassembly rather than inhibiting ciliogenesis. Data from two independent experiments.

Given the presence of Fbxo41 at neuronal centrioles (Figs 1d and 2e), we tested whether Fbxo41 plays a role in the regulation of cilia structure. Primary neurons were infected with shRNAs or EGFP-tagged Fbxo41 constructs at day *in vitro* 1 (DIV1, Fig. 3a), and cilia length was measured at DIV15 (Fig. 3b) using the endogenous ciliary marker Type III adenylyl cyclase (ACIII)^36^. Fbxo41 silencing was effective (Fig. 3c) but had no effect on cilia length or the percentage of ciliated neurons, indicating that Fbxo41 is not essential for neuronal ciliogenesis (Fig. 3d–f). However, Fbxo41^WT^ expression in wild type neurons drastically reduced cilia length as well as the percentage of ciliated neurons (Fig. 3d–f). To discriminate whether elevated levels of Fbxo41 impair ciliogenesis or promote cilia disassembly (Fig. 3b), we also performed these experiments at a later time point. Neurons infected after ciliogenesis (DIV13)^15^ and fixed at DIV21 showed the same reduction in cilia length (Fig. 3f), suggesting Fbxo41 expression promotes cilia disassembly rather than inhibiting ciliogenesis (Fig. 3b). Similar results were obtained in human retinal pigment epithelial cells (hTERT-RPE1). Using either Arl13b or acetylated tubulin as cilia marker, we observed a significant decrease in ciliation upon expression of Fbxo41^WT^ (Supplementary Fig. S4). Fbxo41 expression or silencing in high-density neuronal cultures did not affect total protein levels of ACIII or Arl13b, verifying that the decreased length measured with these ciliary markers was not due to a reduction in their expression levels (Fig. 3c). Taken together, using two independent model systems and three different cilia markers, we demonstrate that increased Fbxo41 expression robustly disassembles primary cilia.

### Centrosomal levels of Fbxo41 correlate with cilia length

**Figure 4:**
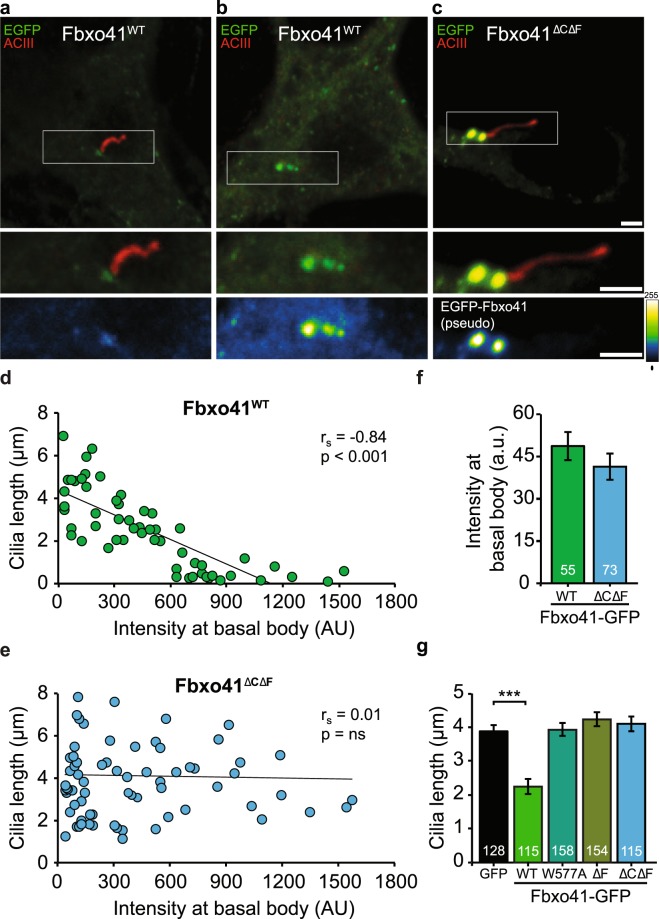
Cilia length inversely correlates with centriolar Fbxo41 levels and requires a functional F-box domain. (**a**–**c**) Primary hippocampal neurons were infected with the indicated constructs at DIV1, fixed at DIV15, and immunostained with GFP (green), ACIII (red) and MAP2 (blue) antibodies. Fbxo41^WT^, but not Fbxo41^ΔCΔF^ mutant levels at centrosome correlate with cilia length: (**a**) neuron with low Fbxo41^WT^ expression (green) and long cilia (red), (**b**) neuron with high Fbxo41^WT^ expression and small/no cilia, and (**c**) neuron with high expression of Fbxo41^ΔCΔF^ mutant and a long cilium. The bottom panel is a pseudo color image of EGFP-Fbxo41 to emphasize the difference in Fbxo41 expression. Calibration bar illustrates color-coded signal intensity. Scale bars, 2 μm. (**d**) Cilia length inversely correlates with centriolar Fbxo41 levels. Scatterplot displays the relationship between Fbxo41^WT^ intensity at the centrioles and cilia length. Each dot represents a single measurement from an individual neuron (n = 55 cells). The black line shows a linear fit through the data. Inset displays the Spearman correlation coefficient (rs) and significance (p-value). (**e**) Cilia length does not correlate with centriolar Fbxo41^ΔCΔF^ levels. Scatterplot displays the lack of relationship between Fbxo41^ΔCΔF^ intensity at the centrioles and cilia length. Each dot represents a single measurement from an individual neuron (n = 73 cells). The black line shows a linear fit through the data. Inset displays the Spearman correlation coefficient (r_s_) and significance (p-value). (**f**) Similar mean centriolar intensity between neurons infected with Fbxo41^WT^ or Fbxo41 ^ΔCΔF^. Data from (**d**,**e**). Mann-Whitney Test, p = 0.155. (**g**) Cilia length was reduced by Fbxo41^WT^, but not by Fbxo41 with a deleted or mutated F-box domain. Kruskal-Wallis Test, *** p < 0.001. Data was pooled from two independent experiments. Data are represented as mean ± SEM.

To further investigate the role of Fbxo41 in ciliary disassembly, we tested whether Fbxo41 levels at the centrosome correlate with cilia length (Fig. 4a–c). Indeed, neurons with low Fbxo41^WT^ expression at centrosomes had normal cilia length (Fig. 4a), whilst neurons with high levels of Fbxo41^WT^ at centrosomes generally had no visible cilia (Fig. 4b). We quantified Fbxo41^WT^ levels at the centrosome and detected a significant inverse correlation between cilia length and centrosomal Fbxo41^WT^ expression (Fig. 4d). The fact that cilia length gradually declines with Fbxo41^WT^ intensity is compatible with a model in which cilia are disassembled, instead of a binary model such as cilia shedding. This observation raised the possibility that Fbxo41 promotes cilia disassembly by sequestering, hindering or competing for proteins locally at centrioles. For this reason, we also tested the effect of Fbxo41^ΔCΔF^ (Fig. 4c), which does target to centrioles but lacks the F-box domain essential for SCF-complex assembly (Fig. 2g). No correlation between cilia length and centrosomal expression was observed for Fbxo41^ΔCΔF^ (Fig. 4e), despite the fact that Fbxo41^ΔCΔF^ targets to centrosomes (Figs 2e and 4f) and showed similar expression levels compared to Fbxo41^WT^ (Figs 2e and 4e,f). The fact that expression of Fbxo41^ΔCΔF^ had no effect on cilium length, shows that a functional F-box domain is required for the dosage-dependent relation between centrosomal Fbxo41 levels and neuronal cilia length. To further explore the mechanism by which Fbxo41 reduces cilia length, we infected primary neurons with various Fbxo41 mutants lacking a functional F-box domain (Fig. 4g). Unlike Fbxo41^WT^, expression of Fbxo41^ΔF-box^ or Fbxo41^W577A^ had no effect on cilia length (Fig. 4g). Collectively, these results demonstrate that Fbxo41 requires a functional F-box domain for centriole targeting and cilia disassembly.

### Phorbol esters, but not neuronal activity, affects neuronal cilia length

**Figure 5:**
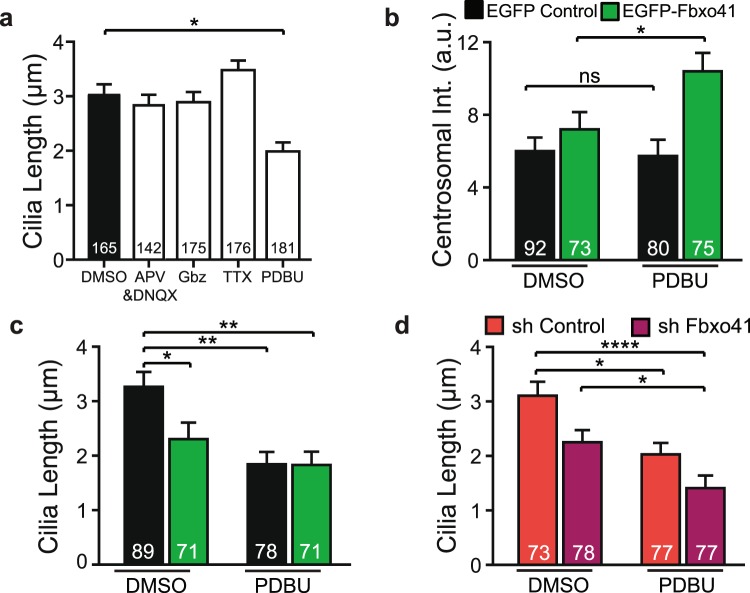
Phorbol esters, but not neuronal activity, promote ciliary disassembly. (**a**) In neurons at DIV 16 cilia length is unchanged by decreasing (using APV & DNQX or TTX) or increasing neuronal activity (using Gbz), but it is significantly lower in neurons treated with Phorbol ester (PDBU). Data from five independent experiments. (**b**,**c**) At DIV 16 the decrease in cilia length caused by 6 h treatment with PDBU correlates with an increase in centrosomal accumulation of Fbxo41. Data from two independent experiments. (**d**) Neurons treated at DIV 11 with PDBU also show a decrease in ciliary length. This decrease is also observed in neurons lacking Fbxo41 (expressing shRNA against Fbxo41 since DIV1). **p < 0,01 ****p < 0,0001 Kruskal-Wallis Test, followed by a post-hoc Dunn’s multiple comparisons test. Data are represented as mean ± SEM.

As the mechanisms that modulate cilia length in neurons remain elusive, we set out to identify physiological stimuli that can induce cilia disassembly. As such, we tested if altering neuronal activity or modulating synaptic pathways in high density cultures induces cilia re-modeling. The stimuli we used to modulate neuronal activity had no effect on cilia length: inhibiting post-synaptic activity by blocking NMDA and AMPA receptors (using APV and DNQX, respectively), inhibiting neuronal activity (using Tetrodotoxin) or increasing network activity (using the GABA-A receptor antagonist Gabazine) for 48 hours had no effect on cilia length (Fig. 5a). Hence, changes in neuronal network activity do not affect cilia morphology. However, treating neurons with the diacylglycerol analog PDBU, an activator of protein kinase C (PKC), a well-known modulator of synaptic plasticity^37^, decreased cilia length (Fig. 5a). Importantly, the PDBU-induced reduction in cilia length correlated with increased centrosomal expression of Fbxo41-EGFP (Fig. 5b,c). The effect of PDBU and Fbxo41 on cilia length were not additive (Fig. 5c), indicating that PDBU and Fbxo41 may act on a similar pathway. In order to test if Fbxo41 is required for PDBU-dependent cilia disassembly, we tested if PDBU also reduced cilia length in the absence of Fbxo41 (Fig. 5d). DIV11 neurons treated with PDBU showed a decrease in cilia length in the presence or absence of Fbxo41 (Fig. 5d). PDBU induces cilia disassembly and increased Fbxo41 centrosomal expression in cultured neurons, but PDBU-dependent cilia disassembly is not dependent on Fbxo41.

### Fbxo41-dependent cilia disassembly mechanisms differ between mitotic cells and neurons

**Figure 6:**
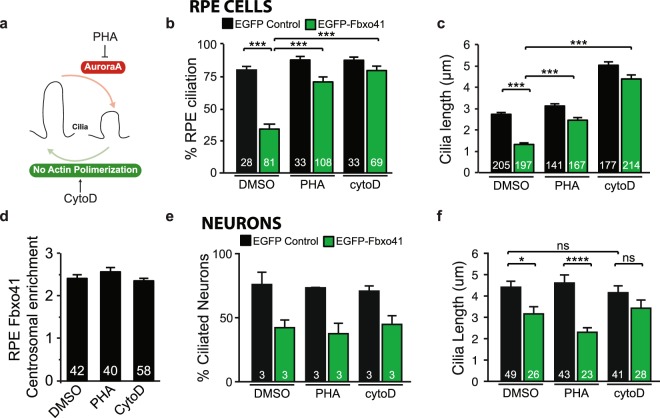
Fbxo41-dependent cilia disassembly mechanisms differ between mitotic cells and neurons. (**a**) Scheme showing previously described effects of AuroraA kinase and actin on cilia integrity in RPE1 cells. AuroraA kinase is required for ciliary disassembly, and inhibition with PHA prevents ciliary disassembly in mitotic cells^14^. In addition, inhibiting actin polymerization with CytoD, increases ciliary length^37^. (**b**-**d**) RPE1 cells were infected with EGFP or EGFP-Fbxo41^WT^, and treated with either DMSO, PHA-680632 (0.5 μM) or CytoD (0.5 μM) for 18 h. Fbxo41-mediated decrease in cilia length and percentage of ciliated cells was rescued by inhibition of Aurora A kinase and inhibition of actin polymerization. (**d**) Ratio of centrosomal over cytoplasmic Fbxo41 intensity quantified in EGFP-Fbxo41^WT^ expressing RPE1 cells for each experimental condition in (**h**). No difference in centrosomal enrichment was observed. (**e**) Neurons were infected with EGFP or EGFP-Fbxo41^WT^, and treated with either DMSO, PHA-680632 (0.5 μM) or CytoD (0.5 μM) for 48 h. Fbxo41-mediated decrease in percentage of ciliated neurons was not rescued by inhibition of Aurora A kinase or inhibition of actin polymerization. (**f**) Cilia length (in cells containing cilia) in EGFP-Fbxo41^WT^ expressing neurons was rescued by CytoD but not PHA-680632. Note that in neurons CytoD does not increase cilia length. Data from two independent experiments. Kruskal-Wallis Test, ***p < 0.001 ****p < 0.0001. Data are represented as mean ± SEM.

Cilia have been extensively studied in mitotic cells, where both Aurora A kinase and actin have emerged as key regulators of cilia structure^14,38^. Aurora A is a centrosomal kinase that plays a pivotal role in cilia disassembly, whereas the absence of actin polymerization increases cilia length and numbers (^14,38^ and Fig. 6a). We tested whether Fbxo41 promotes cilia disassembly through Aurora A kinase activation by expressing Fbxo41^WT^ in RPE1 cells in the presence of Aurora A inhibitor PHA-680632 (Fig. 6b,c). As described above, cilia length and number were reduced by Fbxo41^WT^ expression (Fig. 6b,c). However, in mitotic cells Fbxo41-mediated cilia disassembly was rescued by inhibiting Aurora A kinase activity (Fig. 6b,c). Drugs that perturb rearrangements of the actin-cytoskeleton also have a strong effect on cilia structure^38,39^, and these drugs have been shown to rescue disrupted cilia structure in several *null* mutants^38–42^. We assessed the importance of the actin-network in Fbxo41-mediated cilia disassembly by treating RPE1 cells and neurons with Cytochalasin D (CytoD), an actin filament polymerization inhibitor. In control RPE cells expressing only GFP, CytoD increased ciliary length, without affecting the percentage of ciliated cells. In Fbxo41 expressing RPE cells, CytoD rescued cilia length and percentage of ciliated cells (Fig. 6b,c). In neurons, the effect of CytoD and Aurora A Kinase on cilia length has not been studied. In contrast to its effect in mitotic cells, CytoD does not increase cilia length in neurons (Fig. 6f). However, CytoD treatment of Fbxo41-expressing neurons rescued cilia length, but not percentage of ciliated neurons, to DMSO control neurons (Fig. 6e,f). Inhibiting Aurora A kinase failed to rescue the percentage of ciliated neurons and cilia length in Fbxo41-expressing neurons (Fig. 6e,f). Neither of the drugs did affect Fbxo41 targeting to centrosomes (Fig. 6d), indicating that the rescued cilia structure is not due to displacement of Fbxo41 from centrosomes. Moreover, these data show that centrosomal targeting of Fbxo41 occurs independently of Aurora A kinase activity and the actin-cytoskeleton. Overall, our results indicate that rearrangements of the actin-cytoskeleton are required for Fbxo41 mediated cilia disassembly, and that ciliary disassembly occurs via different mechanisms in mitotic cells and neurons.

### Fbxo41-dependent cilia shortening impairs sonic hedgehog signaling

**Figure 7:**
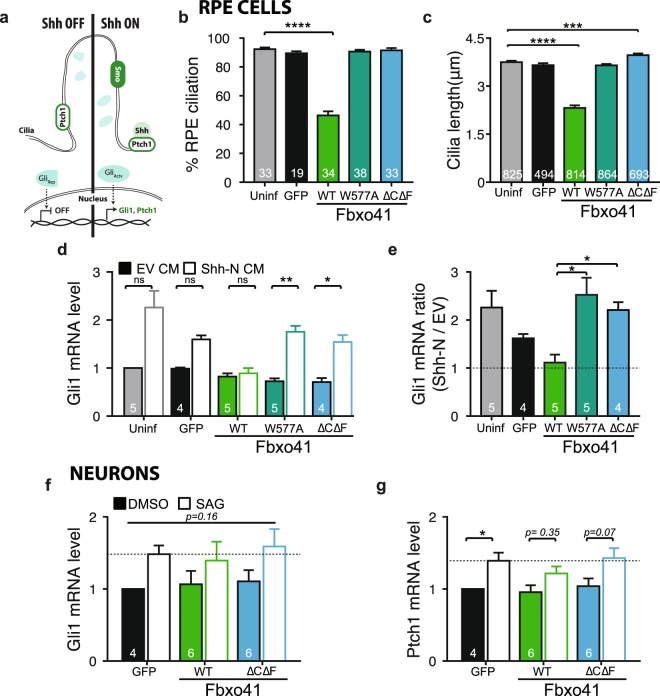
Fbxo41 regulates sonic hedgehog signaling. (**a**) Scheme depicting sonic hedgehog cascade, which in mammals concentrates in the cilium^3^. In the basal state, the Ptch1 receptor is in the cilium, and inhibits Smo targeting to cilium. Upon binding of sonic hedgehog (Shh) to Ptch1, Smo can target to the cilium and induces the conversion of the Gli proteins from a repressed (Gli_Rep_) to an activated (Gli_Actv_) form. Once activated, Gli proteins target to nucleus and promote transcription of Gli1 and Ptch1^43^. (**b**–**e**) RPE1 cells were infected with the indicated constructs for three days and then fixed for quantification of percentage of ciliated cells (**b**) and cilia length (**c**), or treated with Shh-conditioned media to study sonic hedgehog cascade activation (**d**,**e**). (**b**) Cells expressing Fbxo41 had significantly less cilia than the cells in other conditions. Data from 2 experimental weeks. (**c**) Cilia length determined in the same cells as in (**b**), showed significantly smaller cilia in cells expressing Fbxo41^WT^ but not in cells expressing Fbxo41^W577A^ or Fbxo41 ^ΔCΔF^. (**d**) Fbxo41 overexpression prevents the increase in Gli1 expression in cells stimulated with Shh-N condition media. Data from 4 or 5 experimental weeks. (**e**) The ratio of Gli1 mRNA levels between cells stimulated with Shh-N CM and control condition media is significantly lower in cells overexpressing Fbxo41, compared to cells expressing GFP, other Fbxo41 mutants and uninfected cells (same cells as in (**d**)). (**f**) Gli1 mRNA level increase in response to SAG treatment is not statistically significant in any condition. (**g**) Neurons expressing GFP but not those expressing Fbxo41, show increased Ptch1 mRNA levels in response to SAG treatment. Data are represented as mean ± SEM. *p < 0.05, **p < 0.01, ***p < 0.001 ****p < 0.0001 Kruskal-Wallis test followed by a Dunn’s multiple comparison post-hoc test

After establishing the role of Fbxo41 on cilia structure, we investigated its functional consequences, by studying Sonic hedgehog (Shh) signaling, a ciliary pathway^3,4^. At the basal state, the Shh receptor Ptch1 is in the cilium. Upon Shh binding, Ptch1 releases its inhibition of Smo, allowing it to target to the cilium. There Smo allows Gli proteins to go from a repressor to an activated form, promoting the expression of target genes such as Gli1 and Ptch1 (^43^, and Fig. 7a). To study the effect of Fbxo41 on the Shh cascade, we expressed GFP, or Fbxo41 constructs in RPE1 cells and neurons, activated the Shh cascade, and assessed Ptch1 and Gli1 expression. Fbxo41^WT^, but not Fbxo41^W577A^ and Fbxo41^ΔCΔF^, reduced the percentage of ciliated RPE1 cells (Fig. 7b and Supplementary Fig. S4), and cilia length (Fig. 7c), as observed before. RPE1 cells were treated with conditioned media (CM) from HEK293T cells expressing N-terminal Shh (Shh-N) to activate the Shh cascade, or an empty vector (EV) as control. Shh cascade activation resulted in a significant increase in the levels of Gli1 mRNA levels in control cells and cells expressing Fbxo41^W577A^ or Fbxo41^ΔCΔF^, but not in cells expressing Fbxo41^WT^ (Fig. 7d). In addition, the ratio of Gli1 expression between cells treated with Shh-N and EV was significantly lower in Fbxo41 expressing cells (Fig. 7e). To assess if the Shh cascade is also affected in neurons in the presence of Fbxo41, we activated the cascade using the Smoothened agonist SAG, as neurons did not tolerate HEK293T CM, and measured mRNA levels of Ptch1 and Gli1 (Fig. 7f,g). Shh pathway activation with SAG in neurons was much less efficient than Shh-N in RPE cells and Gli1 mRNA levels were not significantly upregulated upon SAG treatment in all groups (Fig. 7f). The other major downstream Shh gene Ptch1 was significantly upregulated upon SAG treatment in GFP expressing neurons, but not in those expressing Fbxo41 (Fig. 7g). Ptch1 response in neurons expressing Fbxo41^ΔCΔF^ did not reach statistical significance. Collectively, these data indicate that Fbxo41-indcued cilia disassembly affects their signaling capacity in mitotic cells and, albeit to a lesser extent, in neurons.

## Discussion

Although the presence of primary cilia in most neuronal cell types is now well established, and it is widely recognized that defective cilia signaling contributes to brain dysfunction, many questions about the structural regulation of these dynamic organelles remain. We identified a neuron-specific F-box protein, Fbxo41, which assembles into SCF-complexes and targets to centrosomes. Its accumulation in centrosomes promotes disassembly of neuronal primary cilia and affects their signaling capacity.

Like other F-box proteins, Fbxo41 assembles into SCF-complexes by interacting with Skp1 and Cul1 through its F-box domain (Fig. 1a and Supplementary Fig. S1). Interestingly, deleting (Fbxo41^ΔF^) or mutating (Fbxo41^W577A^) the F-box domain abolishes SCF-complex formation as well as centrosomal targeting. Skp1 and Cul1 are highly enriched at centrosomes^20^, but interaction with these proteins is insufficient for centrosomal targeting of Fbxo41, since truncation mutants containing an intact F-box domain but lacking the Coiled-coil domain (Fbxo41^ΔCC^ and Fbxo41^RH^) do not enrich at centrosomes (Fig. 2 and Supplementary Fig. S3). Interestingly, the F-box domain is not required for centrosomal targeting in a mutant lacking the C-terminal domain (Fbxo41^ΔCΔF^). Therefore, our data support a mechanism whereby the C-terminal domain of Fbxo41 functions to inhibit centrosome targeting, probably through steric hindrance of the Coiled-coil domain, and the F-box domain releases this inhibition, likely through Skp1-dependent mechanisms.

Centrosomal expression of Fbxo41 robustly reduced primary cilia length (Figs 3, 4, 7 and Supplementary Fig. S4). Mutants impaired in SCF-complex formation, Fbxo41^ΔF-box^, Fbxo41^W577A^ and Fbxo41^ΔCΔF^, fail to promote cilia disassembly (Fig. 4), indicating that Fbxo41 requires engagement into- and possibly ubiquitin ligase activity of- SCF-complexes to promote cilia disassembly. Importantly, Fbxo41^ΔCΔF^ does target to centrioles, but increased centrosomal levels of Fbxo41^ΔCΔF^ do not reduce cilia length, which indicates that the effect of Fbxo41 on cilia length is not a result of build-up of protein at the centrosome but requires functional Fbxo41. Similar mechanisms have been shown for other centrosomal proteins, as overexpression of active Plk1 reduces the percentage of ciliated cells, but not overexpression of kinase dead Plk1^44^. Similarly, overexpressed wildtype and catalytically dead Nek2 target to centrosomes, but only wildtype Nek2 reduces cilia length^45^. Hence, our data shows that the engagement of Fbxo41 in SCF complexes, and not its centriolar targeting per se, affects cilia size. This is in line with reports that implicate the ubiquitin system in disassembly of cilia^23,46^. Although Fbxo41 expression in the brain is restricted to neurons (Fig. 1c and^25^), we also observed cilia disassembly in non-neuronal cells upon overexpression of Fbxo41 (Fig. 7 and Supplementary Fig. S4), suggesting Fbxo41-mediated cilia disassembly in neurons may occur through ubiquitination of conserved cilia-associated proteins.

Recent work in RPE1 cells has revealed proteins involved in cilia disassembly. These include AuroraA, Plk1, HDAC6, Tctex1, and Nek2^14,42,44,45,47,48^. None of these proteins are required for ciliogenesis but overexpression induces robust disassembly of primary cilia. We found similar phenotypes for Fbxo41 in neurons, where Fbxo41 silencing (Figs 3 and 5) or expression of dominant-negative mutants (Fig. 4g) does not disrupt ciliogenesis or ciliary maintenance, but overexpression promotes cilia disassembly. Clearly, maintaining cilium size (and hence its signaling capacity) is essential for cellular viability, which may explain why the structure of this organelle is maintained upon depletion of a single centrosomal protein.

Accumulation of catalytically-active Fbxo41 at centrioles is sufficient to disassemble neuronal primary cilia, but can ciliary disassembly in neurons occur in the absence of Fbxo41? In contrast to well-studied mechanisms of cilia disassembly in mitotic cells, mechanistic insight in cilia disassembly in post-mitotic neurons is generally lacking. We therefore tested several stimuli that could promote ciliary disassembly in neurons and observed that PKC activation by phorbol esters reduced cilia length, while modulation of neuronal network activity did not. The latter implies that neuronal cilia maintain their signaling capacity independent of network activity status. This robustness may be indicative of the importance of cilia signaling for neuronal function. In fact, to our knowledge in addition to the effects of PDBU reported here only brain ischemia results in cilia length reductions^49^. Although there was no additive effect of PDBU on Fbxo41-mediated cilia shortening, PDBU-dependent cilia length reduction in neurons was independent of Fbxo41. Hence, although increased centriolar Fbxo41 levels leads to disassembly of cilia, Fbxo41 is not required during PDBU induced ciliary disassembly in neurons. This indicates that PDBU also triggers Fbxo41-independent pathways to reduce cilia size.

How does Fbxo41 promote disassembly of primary cilia? In dividing cells, timely disassembly of cilia is required for liberation of the centrioles and mitotic spindle formation. The molecular mechanisms underlying cilia disassembly are not completely understood but in mitotic cells involve Aurora A kinase activation^14,50,51^, intraflagellar transport^14^, rearrangements of the actin cytoskeleton^38,39^ and/or modulation of (axonemal) microtubule stability^14^. CytoD blocks rearrangements of the actin cytoskeleton and increases cilia length in several cell types^38–42^, but not in neurons (Fig. 6). CytoD increased cilia length in RPE1 cells in both control and Fbxo41-GFP expressing cells, indicating that this mechanism is not affected by Fbxo41. In neurons, CytoD rescued Fbxo41-mediated ciliary shortening without affecting cilia length in control neurons. Together these data argue for a role of actin polymerization in the mechanism of Fbxo41-dependent ciliary disassembly. Aurora A is a centrosomal kinase that plays a pivotal role in cilia disassembly during the cell cycle. Aurora A, which itself is activated through many different pathways^14,47,50,51^, phosphorylates HDAC6, which in turn deacetylates and destabilizes axonemal microtubules^14^. We found that Fbxo41 promotes cilia disassembly through activation of the Aurora A pathway in RPE cells, but not in neurons, and that Fbxo41 expression leads to a decrease in acetylated axonemal microtubules (Supplementary Fig. S4). This suggests that there are important differences between mechanisms regulating ciliary structure in mitotic cells and post-mitotic neurons. Further elucidation of these differences is pivotal for understanding ciliopathies and their effects on the brain.

Importantly, we found that Fbxo41 regulation of ciliary length impacts Shh signaling, a canonical ciliary pathway in mitotic cells. RPE1 cells and, albeit to a lesser extent, neurons respond to Shh cascade activation by increasing mRNA levels of Gli1 and Ptch1 respectively, but not in the presence of Fbxo41. This indicates that cilia length impacts ciliary signaling. In line with the notion that defective cilia signaling in neurons contributes to brain dysfunction, a recent study showed that deletion of Fbxo41 affects neuronal migration of granule neurons in the developing cerebellum^25^. Future studies will address the question whether defective cilia signaling in the absence of Fbxo41 underlies these defects in cerebellar development.

Overall, our study sheds light on a molecular mechanism that controls primary cilia structure and signaling in neurons and highlights potential differences in cilia size modulation in mitotic and post-mitotic cells. We describe a role for Fbxo41 in this process and show that this requires rearrangements of the actin cytoskeleton in mitotic and post-mitotic cells. Primary cilia and Shh signaling have been shown to be important for crucial processes such as brain development and regulation of neural stem cell populations. By unraveling a new process through which neural cilia can be regulated this study contributes to a better understanding of these processes, and its implications in disease.

## Materials and Methods

### Laboratory animals

Animal experiments were approved by the animal ethical committee of the VU University/VU University Medical Centre (license number: FGA 11-03) and are in accordance with Dutch governmental guidelines and regulations.

### Cell culture and transfection

Primary neurons were prepared from embryonic day 18 mice as previously described^52^. Hippocampi were dissected in Hanks Buffered Salt Solution (Sigma-Aldrich) buffered with 7 mM HEPES (Invitrogen) and digested with 0.25% trypsin (Invitrogen) for 20 minutes at 37 °C. Hippocampi were washed and triturated with fire-polished Pasteur pipettes, counted, and plated in Neurobasal medium (Invitrogen) supplemented with 2% B-27 (Invitrogen), 1.8% Hepes, 0.25% GlutaMAX (Invitrogen), and 0.1% penicillin-streptomycin (GE Healthcare). High-density cultures (25,000 or 600,000 neurons/well) were plated on pregrown cultures of rat glia cells (25,000 or 50,000 cells/well) on 18 mm glass coverslips or directly onto Poly-L-Ornithine coated plastic for 12-well plates (for imaging experiments) or 6-well plates (for biochemical experiments), respectively.

HEK293T and hTERT-RPE1 (gift from Rob Wolthuis, VUmc, Amsterdam) cells were cultured in DMEM (Gibco) supplemented with 10% fetal bovine serum (Gibco), penicillin-streptomycin and non-essential amino acids (Gibco). HEK293T cells were transfected at ~70% confluency in serum free DMEM using the calcium phosphate method^53^. RPE1 cells were infected with lentivirus in serum free medium. All DNA vectors used were from mouse origin. HEK293T cells were cultured on Poly-D Lysine (Sigma) coated 18 mm glass coverslips (for imaging experiments) or directly on plastic (for biochemical experiments).

### Immunoprecipitation experiments

Forty hours post-transfection, HEK293T cells were washed in ice-cold PBS, harvested in Lysis buffer (50 mM Tris pH 7.5, 1% Triton X-100, 1.5 mM MgCl_2_, 5.0 mM EDTA, 100 mM NaCl_2_ and phosphatase/protease inhibitor cocktails (Santa Cruz and Sigma)) and tumbled for 1 hour to ensure complete lysis. The mouse brain was collected and immediately lysed in Lysis buffer. Lysates were then centrifuged at 10,000xg for 10 minutes and pellets were discarded. Bradford assays were performed to determine protein concentrations. Antibodies were added to lysates and gently tumbled overnight. Agarose beads (Vector Laboratories) were washed twice in Lysis buffer, added to the samples, and tumbled for 1 hour. Samples were spun down at 4000xg for 5 minutes and washed in Wash buffer (50 mM Tris, 0.1% Triton X-100, 1.5 mM MgCl2, 5.0 mM EDTA, 250 mM NaCl_2_ and phosphatase/protease inhibitor cocktails) three times. Lysates were kept on ice or at 4 °C throughout the entire experiment. Beads were boiled for 10 minutes in 2xLSB and samples were run on SDS-PAGE.

### Generation of shRNA lentivirus particles

Fbxo41 shRNA target sequences were selected using iRNAi (Mekentosj, v2.0) and cloned into the pLL3.7 backbone^54^ with a Synapsin promoter driving EGFP expression. Oligonucleotides were designed with target sequences separated by a seven-nucleotide loop^55^ and a XhoI digestion site overhang. Fbxo41 shRNA#1;

5′-TGCTGCCCTCTTCTGTATCTTTCAAGAGAAGATACAGAAGAGGGCAGCTTTTTTC.

Fbxo41 shRNA#2;

5′-TAGGCTCACTCGTTAACATTTTCAAGAGAAATGTTAACGAGTGAGCCTGGTTTTTTC (shRNA target sequences are underlined).

### Immunocytochemistry

Cultured cells were fixed at indicated ages with 3.7% formaldehyde in PBS, pH 7.4, for 10 minutes at room temperature. For some of the experiments with centriolar antibodies, cells were fixed with an ice-cold Methanol and Acetone solution (1:1) at −20 °C for 10 minutes. Cells were washed three times in PBS, permeabilized in 0.1% Triton X-100 (Sigma-Aldrich) in PBS for 10 minutes, and incubated with blocking solution (PBS containing 2% normal goat serum, and 0.1% Triton X-100) for 40 minutes at room temperature. Cells were then incubated with primary antibodies in blocking solution for 2 hours, washed three times with PBS, and incubated with secondary antibodies conjugated to Alexa Fluor in blocking solution for 1 h at room temperature (1:1000; Invitrogen). Primary antibodies used were rabbit polyclonal ACIII (1:1000; Encor), rabbit polyclonal Arl13b (1:500; ProteinTech), rabbit polyclonal Cep135 (1:500, Sigma), rabbit polyclonal Fbxo41 (1:500; Synaptic Systems, 6897), mouse monoclonal FLAG (1:4000; Sigma), rabbit polyclonal GFP (1:2000; GeneTex), chicken polyclonal GFP (1:2000; AVES), mouse monoclonal HA (1:1000; Roche), chicken polyclonal MAP2 (1:20,000; Abcam), mouse monoclonal Myc (1:1000; Roche), mouse monoclonal Pericentrin (1:1,500; BD Transduction Laboratories), mouse monoclonal γ-tubulin (1:500; Sigma, clone GTU-88), rabbit polyclonal EEA1 (1:100; CellSignaling), mouse monoclonal GM130 (1:1000; Transduction Labs), rabbit polyclonal LIMPII (1:200; Novus Biologicals), polyclonal rabbit Chromogranin B (1:500; SySy), polyclonal guinea pig Synaptophysin1 (1:1000; SySy). Finally, cells were washed three times in PBS and coverslips were mounted in Mowiol (Calbiochem) on glass slides. For visualization of nuclei, TO-PRO3 (1 μM, Invitrogen) was added to the secondary antibody mixture.

### Microscopy

Confocal images were acquired on an LSM 510 Meta Carl Zeiss microscope using a 63× oil objective lens, NA 1.4. Z-stacks were acquired with an interval of 0.5 μm and were maximally projected for analysis and display.

### Image analysis

Experimenter was blind during image acquisition and analysis. Cilia length was measured with ImageJ (National Institutes of Health) plugin Neuron J^56^. Cells were considered ciliated if ciliary structure was longer than 1.3 μm) For each image of RPE cells the percentage of ciliated cells was calculated by dividing the total number of infected ciliated cells, by the total number of infected cells. The efficiency of Fbxo41 was lower, and so more images were analyzed to obtain similar numbers of cells. Centrosomal intensity was quantified by placing circular regions of interest (ROIs) in the centrosome channel. For centrosomal enrichment quantification, for each image, the average intensity of three to five ROIs placed outside the cell was used to determine background intensity. One ROI was placed over the pericentrin or γ-tubulin indicated centrosome and this signal was divided by the average signal of three to six ROIs placed randomly throughout the cytoplasm after background subtraction. Percentage of cells with centriolar enrichment of Fbxo41 mutants was determined by visual scoring. Percentage of ciliated cells was determined by counting the total number of cells in each image, and determining which of those cells were ciliated.

### Two color dSTORM imaging

Prior to staining and imaging, DIV11 hippocampal neurons were infected for 4 to 7 days with Fbox 41^WT^-FLAG. Neurons were fixed in MeOH for 10 minutes, permeabilized for 7 minutes using 0.25% Triton X-100 in PBS, washed and incubated for 30 minutes in blocking solution (2% BSA +0.2% gelatin, 10 mM glycine + 50 mM NH_4_Cl in PBS, pH 7.4). Neurons were incubated with primary and secondary antibodies in the blocking solution. For Fbox 41-FLAG, antibodies against Flag tag (Sigma, clone M2, 1:400 dilution) was used followed by anti-mouse coupled to A647 (Invitrogen, A-21236). For Cep135, rabbit anti Cep135 (Sigma, clone SAB4503685, 1:250 dilution) was used followed by anti-rabbit coupled to A488 (Invitrogen, A-11034). Stained neurons were post-fixed for 10 minutes using 2% PFA in PBS. Imaging was performed using 5 mM MEA, 10% w/v glucose, 700 µg/ml glucose oxidase, 40 µg/ml catalase in PBS. dSTORM microscopy was performed on a Nikon Ti microscope equipped with a 100x Apo TIRF objective (NA. 1.49), a Perfect Focus System, a 2.5x Optovar (to achieve an effective pixel size of 64 nm) and an Andor DU-897D EMCCD camera. Sequential imaging of Alexa Fluor 647 and A488 was performed by continuous oblique laser illumination with 640 nm diode laser and 491 nm DPSS laser, respectively. For A488 the sample was also illuminated with 405 nm diode laser. Between 5000 and 15000 frames were recorded per acquisition with exposure times of 30 ms. Single molecule localization was performed as previously described^57^. A particle table with molecule coordinates and errors was used to reconstruct a super resolution image. 10 nm pixel size was used for super-resolution image display. For two-color imaging, chromatic corrections obtained from images with multichromatic 100 nm-beads (Tetraspeck, Invitrogen) were applied to the A488 particle table. For sample drift during acquisition, a correction algorithm was applied^58^.

### Drug treatments

For modulating neuronal activity high density continental neuronal cultures (40K neurons per well) were treated for 48 h with compounds known to increase or decrease neuronal activity and then fixed at DIV 16 for analysis: Gabazine (Gbz, 10μM), APV (50μM) and 6,7-dinitroquinoxaline-2,3-dione (DNQX, 20μM), tetrodotoxin (TTX; 1μM). For experiments with Phorbol 12,13-dibutyrate (PDBU; 1μM) neurons were treated for 6 hours and fixed at the indicated time points. For experiments testing the effect of AuroraA Kinase and actin in ciliary disassembly RPE1 cells were seeded in DMEM (Gibco) supplemented with 10% fetal bovine serum, and one day later the medium was replaced for DMEM without serum. After 2 days cells were infected with the indicated constructs, and 2 h after treated for 18 h with either DMSO, 0.5μM of PHA-680632 (PHA) or 0.5μM CytoD. Neurons were infected at DIV 9 with the indicated constructs and treated one day later with either DMSO, 0.5μM of PHA or 0.5μM CytoD, and fixed at DIV 12 for analysis. Neurons were infected for one day before treatment with PHA and CytoD.

### Shh Conditioned Media

ShhN and EV condition media were generated as previously described^59^. Briefly, HEK293T cells grown to 70% confluency were transfected (calcium transfection using 1μg DNA, 125 mM CaCl_2_ and HEPES buffered saline) with either empty vector or the N-terminus of rat Shh (gift from Lotte Bang Pedersen, University of Copenhagen). The following day cells were changed to serum free medium, and after 1 day the medium was collected, centrifuged (5 min at 1200 rpm) and filtered (0.2μm filter). The medium was kept at −80 °C and used at 1:1 for 14 to 16 h, as previously described^60^.

### RT-qPCR

RPE1 cells were seeded in DMEM (Gibco) supplemented with 10% fetal bovine serum, and one day later the medium was replaced for DMEM without serum. After 2 days cells were infected with the indicated constructs, and 3 days afterwards treated with conditioned media for 15 hours. Neurons were infected at DIV1 with the indicated constructs and treated with 300–400 nM SAG (Enzo Lifesciences) for 24 h, as done before^61^. Total RNA was isolated using TRIzol reagent (Life Technologies), phase lock gel tubes and ISOLATE II RNA Mini Kit (Bioline) according to manufacturer’s instructions. The purity and quantity of RNA was assessed on a NanoDrop spectrophotometer, and RNA was reversed transcribed into cDNA with SensiFAST^TM^ cDNA Synthesis Kit (Bioline). The resulting cDNA was quantified using the SensiFAST™ SYBR No-ROX Kit (Bioline) in the LightCycler 480 (Roche Life Sciences). Analysis was performed using the Advanced Relative Quantification option in the LightCycler 480 software (the Cp value was determined using the second derivative maximum method). The cDNA was quantified in triplicates, and quantification was only considered valid when the Cp values determined did not differ for more than 1 unit among the triplicates. The relative mRNA quantity was normalized to 18 S in RPE1 cells and EEF in neurons. The primers used are provided in Supplementary Table S1.

### Data representation and statistics

In all graphs, data is presented as mean values ± SEM. Differences between two groups were tested for significance using a Student’s t test for unpaired data when data passed normality test (Kolmogorov-Smirnov) or a Mann-Whitney test when it did not. For multiple group comparisons, one-way ANOVA was used if allowed otherwise the non-parametric Kruskal-Wallis test was used. P-values below 0.05 are considered significant.

## Supplementary information

Supplementary Information

## Data Availability

All the data generated during this work is presented in the paper. Datasets are available from corresponding author upon reasonable request.
