# Supplementary material for: Fbxo41 Promotes Disassembly of Neuronal Primary Cilia
Source: Sci Rep. 2019 Jun 3;9:8179. doi: 10.1038/s41598-019-44589-2 (PMC6546786; doi:10.1038/s41598-019-44589-2)
Supplement: Supplementary file 1 — Supplementary Information [file 41598_2019_44589_MOESM1_ESM.pdf]

Supplementary files

Fbxo41 Promotes Disassembly of Neuronal Primary Cilia

Cillian R. King[1#], Ana R. A. A. Quadros[1#], Anaël Chazeau[3], Ingrid Saarloos[1,2], Anne Jolien van der Graaf[1], Matthijs Verhage[1,2] and Ruud F. Toonen[1*]

[1]Department of Functional Genomics and [2]Department of Clinical Genetics, Center for Neurogenomics and Cognitive Research, VU University Amsterdam and VU Medical Center, 1081 HV Amsterdam, the Netherlands.

[3]Cell Biology, Faculty of Science, Utrecht University, 3584 CH, Utrecht, the Netherlands.

* Corresponding author. Tel: +31 (0) 205986946; E-mail: r.f.g.toonen@vu.nl

# these authors contributed equally

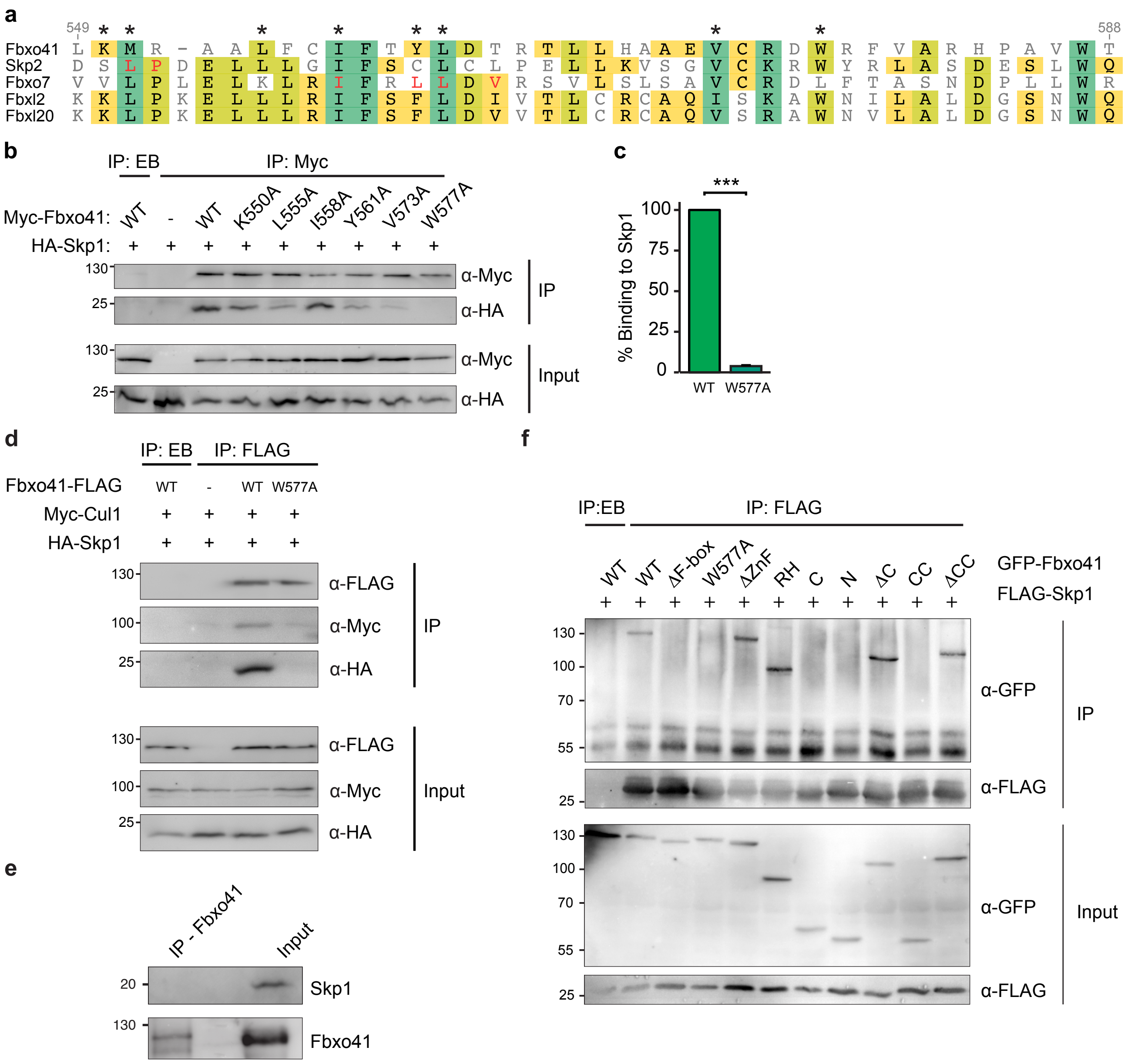

Supplementary Figure S1: Fbxo41 F-box domain mutations disrupt SCF-complex assembly.

(**a**) Sequence alignment highlighting conserved amino acids in the F-box domains of five F-box proteins (made with Geneious, ClustalW, Blosum62, threshold = 1). Numbers indicate amino acid residues in Fbxo41. Amino acids that have previously been shown to ablate Skp1 binding when mutated to Alanine in other F-box proteins are depicted in red[1,2]. Asterisks indicate Fbxo41 residues that were mutated to Alanine in this study.

(**b**) HEK293T cells were transfected with the indicated constructs, immunoprecipitated with empty beads (EB) or Myc antibody and immunoblotted with the indicated antibodies. Of all mutants tested in this experiment, Skp1 binding was most severely abolished in the Fbxo41W577A mutant.

(**c**) Quantification of Skp1 binding from four independent experiments. Skp1 binding to Fbxo41WT was set to 100%. Binding was reduced by 95.9% in Fbxo41W577A. Mann-Whitney Test, *** $p < 0.001$. Error bars represent SEM.

(**d**) Fbxo41 assembles into SCF-complexes regardless of epitope tag size or orientation. HEK293T cells were transfected with the indicated constructs, lysed 40 hours post-transfection and subjected to immunoprecipitation with FLAG antibody. Mutating Fbxo41's F-box domain abolished SCF-complex assembly. In contrast to the experiment depicted in Fig. 1a, where EGFP was fused to the n-terminus of Fbxo41, FLAG was fused to the c-terminus of Fbxo41. Gel was cropped for clarity (full length blot available in Supplementary Fig. S4).
(**e**) Immunoprecipitation of endogenous Fbxo41 from adult brain is not efficient. Both Fbxo41 and Skip1 are present in input, but only a very small amount of endogenous Fbxo41 is immunoprecipitated using Fbxo41 antibodies, not enough to detect Skip1.
(**f**) HEK293T cells were transfected with the indicated constructs (see Fig. 2g) and subjected to immunoprecipitation with empty beads (EB) or FLAG-antibody. Membranes were immunoblotted with indicated antibodies. Only Fbxo41 mutants containing an intact F-box domain immunoprecipitated with Skp1.

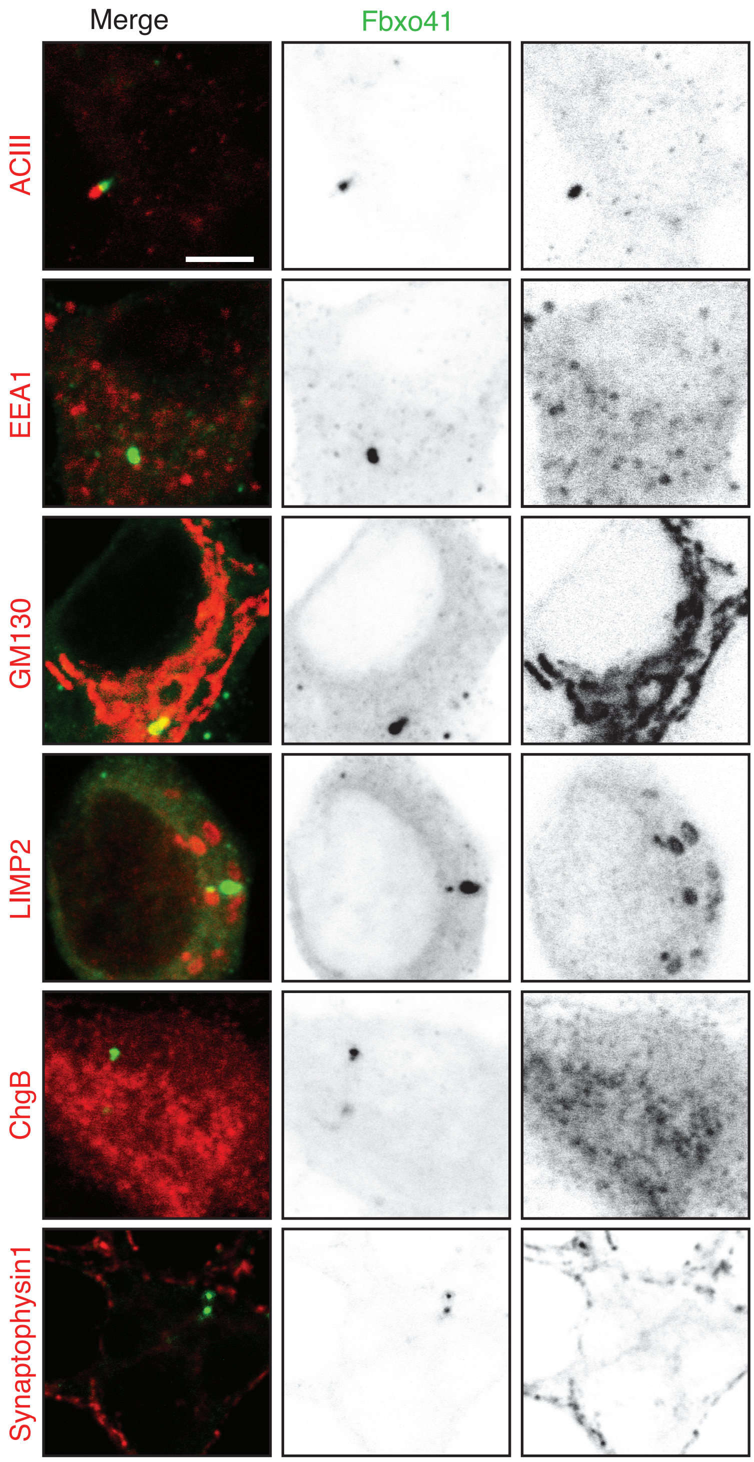

Supplementary Figure S2. Fbxo41 enriches at the ciliary base.
Cortical neurons were infected at DIV 9 with Fbxo41-GFP, fixed 3 days later and stained for several somatic organelles. Neurons were stained with markers for cilia (ACIII), early endosome (EEA1), Golgi (GM130), dense core vesicles (ChgB), and synapses (Synaptophysin1). Although Fbxo41 is not only present at the centrosome, Fbxo41 did not colocalize with any other somatic organelle besides the base of the cilia.

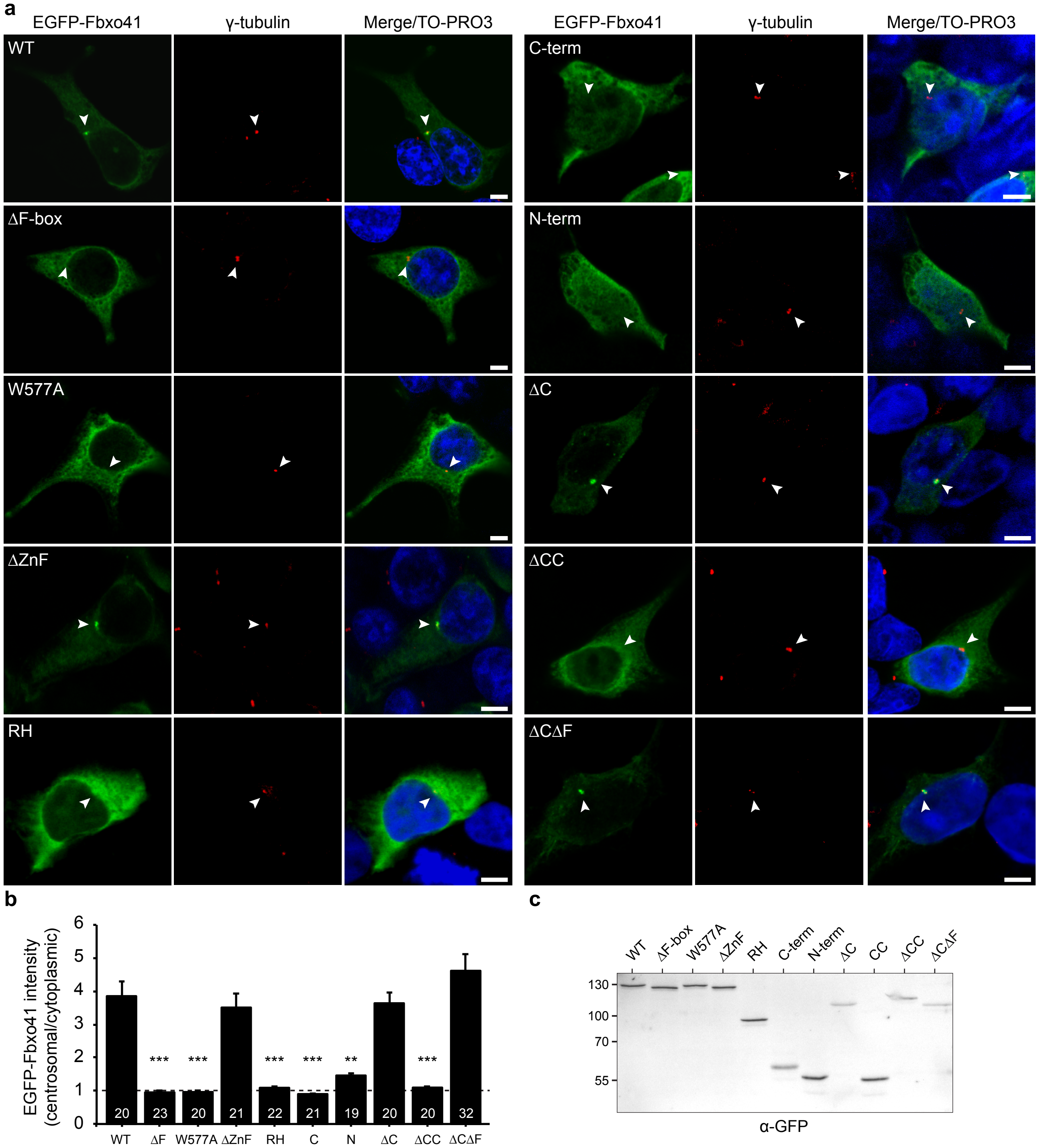

Supplementary Figure S3. The F-box and Coiled-coil domains are essential for centrosome targeting of Fbxo41.

(**a**) HEK293T cells expressing EGFP-fused mutants for domain mapping. Centrosomes were visualized with γ-tubulin antibody (red). TO-PRO3 DNA staining (blue) is included in the merged images. White arrowheads emphasize centrosomes. Cell were fixed 20 hours post-transfection and only cells with low expression levels are depicted. Scale bars, 5 μm.

(**b**) Ratio of centrosomal over cytoplasmic intensity was quantified for each Fbxo41 mutant. Centrosomal enrichment of Fbxo41 was significantly affected in mutants lacking the Coiled-coil domain or mutants in which Skp1 binding was impaired with the c-terminal domain present.

(**c**) Immunoblot of HEK293T cells expressing the indicated EGFP-fused Fbxo41 mutants. Immunoblotting was performed with GFP antibody.

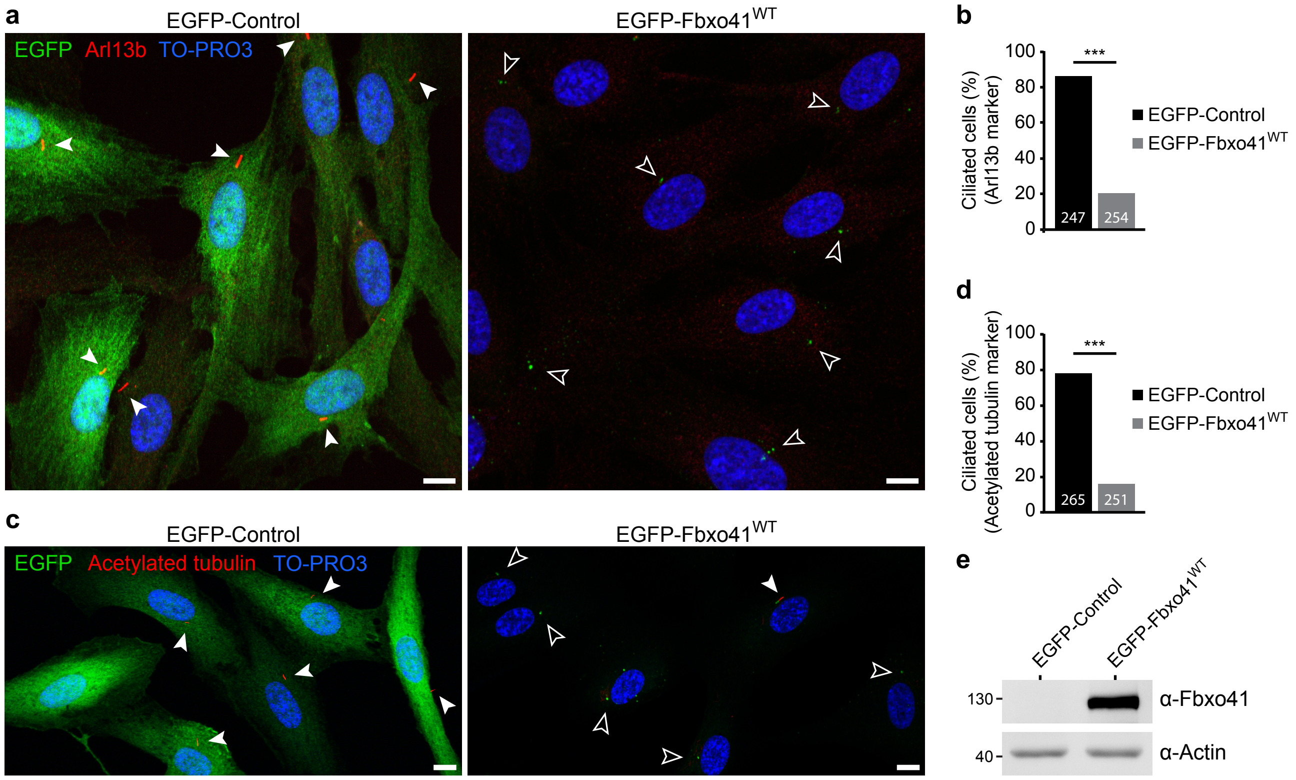

Supplementary Figure S4. Fbxo41 impairs ciliation of RPE1 cells.

(**a**) Typical examples of RPE1 cells infected with EGFP-control or EGFP-Fbxo41WT. Cells were serum starved for 48 hours to induce ciliation. Primary cilia (arrowheads) were visualized with Arl13b antibody (red) and DNA with TO-PRO3 (blue). Like in neurons and HEK-293T cells, EGFP-Fbxo41WT targets to centrosomes of RPE1 cells (open arrowheads). Scale bars, 10 µm.

(**b**) Quantification of ciliation in RPE1 cells from (a). Counting the number of nuclei and dividing this by the number of cilia determined percentage of ciliation. Ciliation differed significantly between EGFP-control (87.9% ciliated, n = 247 cells) and EGFP-Fbxo41 (22.8% ciliated, n = 254 cells) expressing RPE1 cells $\chi 2(1) = 213.8$, *** $p < 0.001$.

(**c**) Typical examples of RPE1 cells infected with EGFP-control or EGFP-Fbxo41WT. Cells were serum starved for 48 hours to induce ciliation. Primary cilia (arrowheads) were visualized with Acetylated-tubulin antibody (red) and DNA with TO-PRO3 (blue). Scale bars, 10 µm.

(**d**) Quantification of ciliation in RPE1 cells from (c). Counting the number of nuclei and dividing this by the number of cilia determined percentage of ciliation. Ciliation differed significantly between EGFP-control (77.7% ciliated, n = 265 cells) and EGFP-Fbxo41 (15.9% ciliated, n = 251 cells) expressing RPE1 cells $\chi 2(1) = 63.7$, *** $p<0.001$.

(**e**) Immunoblot of RPE1 lysates from an experiment performed in parallel with (a and c), demonstrating effective expression of lentiviral constructs. Gel was cropped for clarity (full

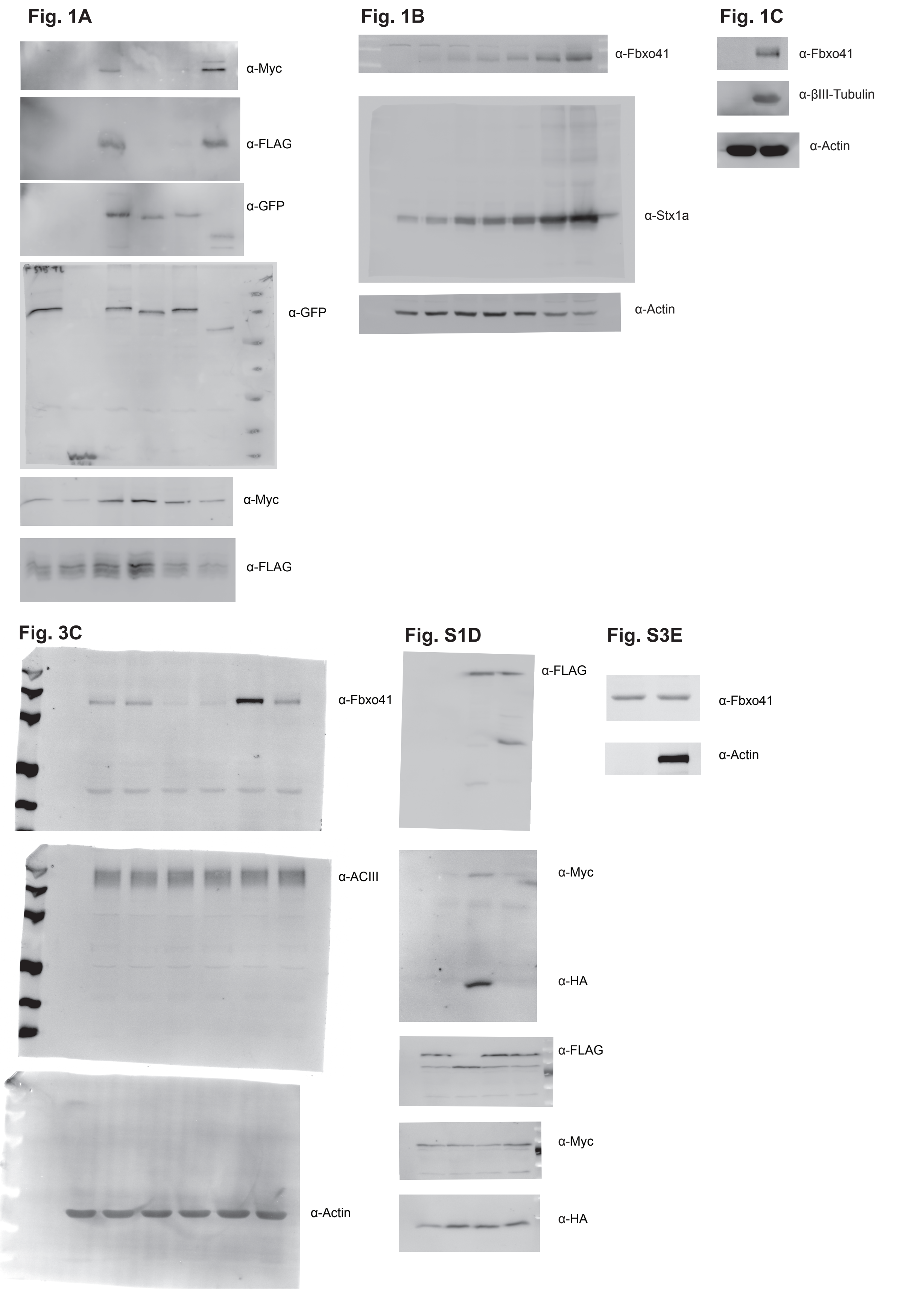

Supplementary Figure S5. Original uncropped blots.
Original blots from Fig. 1a, b and c; Figure 3c; Supplementary Fig. S1d and Supplementary Fig S3e.

Supplementary Table S1: Primers used in qPCR

Primer	Sequence Forward Primer (5'-3')	Sequence Reverse Primer (5'-3')	Cell type	Reference
Gli1	CAGGGAGTGCAGCCAATACAG	GAGCGGCGGCTGACAGTATA	RPE	[3]
18S	AGTCCCTGCCCTTTGTACACA	CGATCCGAGGGCCTCACTA	RPE	[4]
Ptch1	AAAGAACTGCGGCAAGTTTTTG	CTTCTCCTATCTTCTGACGGGT	Neurons	[4]
Gli1	CCAAGCCAACTTTATGTCAGGG	AGCCCGCTTCTTTGTTAATTTGA	Neurons	[4]

References:

1 D'Angiolella, V. *et al.* SCF(Cyclin F) controls centrosome homeostasis and mitotic fidelity through CP110 degradation. *Nature* **466**, 138-142, doi:10.1038/nature09140 (2010).

2 Nelson, D. E. & Laman, H. A Competitive binding mechanism between Skp1 and exportin 1 (CRM1) controls the localization of a subset of F-box proteins. *J Biol Chem* **286**, 19804-19815, doi:10.1074/jbc.M111.220079 (2011).

3 Schou, K. B. *et al.* KIF13B establishes a CAV1-enriched microdomain at the ciliary transition zone to promote Sonic hedgehog signalling. *Nat Commun* **8**, 14177, doi:10.1038/ncomms14177 (2017).

4 Liu, C. *et al.* Regulator of G protein signaling 5 (RGS5) inhibits sonic hedgehog function in mouse cortical neurons. *Mol Cell Neurosci* **83**, 65-73, doi:10.1016/j.mcn.2017.06.005 (2017).
